# Quality of Online Pharmacies and Websites Selling Prescription Drugs: A Systematic Review

**DOI:** 10.2196/jmir.1795

**Published:** 2011-09-30

**Authors:** Grazia Orizio, Anna Merla, Peter J Schulz, Umberto Gelatti

**Affiliations:** ^1^Section of Hygiene, Epidemiology and Public HealthDepartment of Experimental and Applied MedicineUniversity of BresciaBresciaItaly; ^2^Institute of Communication and HealthUniversity of LuganoLuganoSwitzerland

**Keywords:** Internet, pharmaceutical preparations, public health, review, online pharmacies

## Abstract

**Background:**

Online pharmacies are companies that sell pharmaceutical preparations, including prescription-only drugs, on the Internet. Very little is known about this phenomenon because many online pharmacies operate from remote countries, where legal bases and business practices are largely inaccessible to international research.

**Objective:**

The aim of the study was to perform an up-to-date and comprehensive review of the scientific literature focusing on the broader picture of online pharmacies by scanning several scientific and institutional databases, with no publication time limits.

**Methods:**

We searched 4 electronic databases up to January 2011 and the gray literature on the Internet using the Google search engine and its tool Google Scholar. We also investigated the official websites of institutional agencies (World Health Organization, and US and European centers for disease control and drug regulation authorities). We focused specifically on online pharmacies offering prescription-only drugs. We decided to analyze and report only articles with original data, in order to review all the available data regarding online pharmacies and their usage.

**Results:**

We selected 193 relevant articles: 76 articles with original data, and 117 articles without original data (editorials, regulation articles, or the like) including 5 reviews. The articles with original data cover samples of online pharmacies in 47 cases, online drug purchases in 13, consumer characteristics in 15, and case reports on adverse effects of online drugs in 12. The studies show that random samples with no specific limits to prescription requirements found that at least some websites sold drugs without a prescription and that an online questionnaire was a frequent tool to replace prescription. Data about geographical characteristics show that this information can be concealed in many websites. The analysis of drug offer showed that online a consumer can get virtually everything. Regarding quality of drugs, researchers very often found inappropriate packaging and labeling, whereas the chemical composition usually was not as expected in a minority of the studies’ samples. Regarding consumers, the majority of studies found that not more than 6% of the samples had bought drugs online.

**Conclusions:**

Online pharmacies are an important phenomenon that is continuing to spread, despite partial regulation, due to intrinsic difficulties linked to the impalpable and evanescent nature of the Web and its global dimension. To enhance the benefits and minimize the risks of online pharmacies, a 2-level approach could be adopted. The first level should focus on policy, with laws regulating the phenomenon at an international level. The second level needs to focus on the individual. This approach should aim to increase health literacy, required for making appropriate health choices, recognizing risks and making the most of the multitude of opportunities offered by the world of medicine 2.0.

## Introduction

Neo, which pill would you choose? You take the blue pill—the story ends, you wake up in your bed and believe whatever you want to believe. You take the red pill—you stay in Wonderland and I show you how deep the rabbit-hole goes[1].

Although their choice is much less metaphysical than the question posed to Neo in the science fiction movie *The Matrix*, but still important in terms of health care delivery, consumers nowadays can make another decision: they can choose a pill sold at their local pharmacy, as they have always done, or they can choose one from the Web, by purchasing it from a “cyberpharmacy” or “online pharmacy” [2]. An online pharmacy is a company that sells pharmaceutical preparations, including prescription-only drugs, via online ordering and mail delivery, although—as the evidence will show—very few of them behave like a proper “pharmacy” and many of them are not licensed.

The online sale of drugs started in the late 1990s and has expanded so much that the US Food and Drug Administration (FDA) has implemented an entire section on its website dedicated to “Buying medicines over the Internet” [3,4]. The World Health Organization (WHO) is faced with this issue in the context of counterfeit medicines, which “pose a public health risk” [5-7]. Another aspect that is important to consider is that the Internet can facilitate access and thus support abuse of prescription drugs [8].

It is very difficult to estimate the number of online pharmacies and people buying online, the volume of drugs traded, and the revenue and profits generated by such a hidden business. Moreover, the geographical distribution of the phenomenon seems to be very heterogeneous. With regard to the number of online pharmacies, MarkMonitor in a 2009 press release claimed to have found nearly 3000 websites selling prescription medicines, while a 2010 review by the US National Association of Boards of Pharmacy (NABP) investigated the characteristics of 5859 Internet outlets selling prescription medications [9,10]. Regarding access to drugs online, the 2006 Online Health Search, a US survey by the Pew Internet & American Life Project, showed that “prescription or over-the-counter drugs” was the fifth most widely searched health topic on the Internet [11]. Another US telephone survey concluded that 4% of Americans had purchased prescription drugs on the Internet [12]. Very few estimates regarding the revenue and profits of this phenomenon are available, there being great variability in methods and numbers [13-15]. Besides economic aspects, there is a legal issue and jurisdictional consequences: cases of law enforcement acts and legal prosecutions have been reported in the literature [16-18].

This new market has undoubted advantages for patients: access to drugs for the disabled or housebound, access 24 hours a day, a virtually unlimited number of products available, relative privacy, which may encourage patients to ask questions about embarrassing issues, and more affordable prices [19-21]. But direct access to health services, especially drugs, poses a hazard to consumers because it is difficult to determine whether drugs purchased online are counterfeit, unapproved, or illegal [22]. Besides, the inappropriate use of medicines, the limited or nonexistent opportunity for advice (which blurs the line between willful abuse and unknowing misuse), and the risk of increased antibiotic resistance arising from their misuse have also been suggested as negative consequences of online purchase of medication [23,24]. What is more, the chance to circumvent prescription boundaries can be a potential disruptor at several levels, at both an individual and a public health level. At an individual level, this phenomenon can influence the doctor–patient relationship [25,26]. At a public health level, since each country has a unique system, access to drugs from abroad can disrupt the delicate equilibrium that leads to a certain drug price on the basis of taxation, copayment, reimbursement, and negotiation with industry [27].

### Previous Reviews

To our knowledge, 2 reviews about online pharmacies in general and 3 others on specific aspects related to online pharmacies are available. With regard to general reviews, Fung et al [20] searched material published between 1997 and 2002 using 3 scientific databases. They identified 139 articles, although they found that “many of the articles reported about a specific legal case involving an online pharmacy.” Although this review is wide-ranging, it is important to note that it dealt with papers issued 9 or more years ago (2002), a considerable length of time in such a dynamic world as that of the Internet and e-commerce. The most recent review, issued in 2009, is by Nielsen and Barratt [28]. They reviewed the literature on prescription drug misuse through the Internet, focusing on online supply, online monitoring of drug use trends, and electronic prescription monitoring [28]. The part relevant to our review is that on Internet supply; although the work is valuable, the article selection method was not described in detail, making it impossible to establish which databases were screened, with which keywords, and the time of publication. Since the review was submitted for publication on February 5, 2008, we can deduce it explored articles published up to 2007, as confirmed by looking at the references. Besides, the review focused only partially on online supply (referring to only 14 papers in connection with this topic).

Other reviews tackled specific issues but were unable to give an overall picture of the phenomenon. A recent review examined counterfeit phosphodiesterase type 5 inhibitors (PDE5Is) for the treatment of erectile dysfunction, similar to the 2000 review of sildenafil and the Internet [29,30]. The review by Baert and De Spiegeleer gives an overview of the different quality attributes that can be evaluated to gain a complete understanding of the quality of the pharmaceutical product traded on the Internet, as well as the current analytical techniques that serve this objective [31].

### Objectives

This review sought to investigate the available evidence on the phenomenon of online pharmacies. We report data on the 3 main areas on which the literature focuses: the characteristics of the websites, the quality of pharmaceutical products purchased online, and the number of consumers and their characteristics.

## Methods

### Search Strategy

The literature search covered the period up to January 2011. The search was performed on 3 sources: electronic databases, search engines, and institutional websites. First, we searched, without any limitations as to publication date, the following electronic databases: PubMed [32], ISI Web of Knowledge [33], Science Direct [34], and PsycInfo [35]. Second, we searched for gray literature on the Internet using the Google search engine [36] and its tool Google Scholar [37]. We used Google because it is the most widely used search engine [38,39]. Lastly, we investigated the institutional websites of the WHO [40], the WHO European Region [41], the WHO American Region [42], the US Centers for Disease Control and Prevention [43], the FDA [44], the European Centre for Disease Prevention and Control [45], and the European Medicines Agency [46]. We investigated all the results obtained by the databases and the institutional websites but considered only the first 500 results for each keyword appearing in Google and Google Scholar, because the number of relevant articles declined substantially after the first 300 results and because this search engine displays results by relevance using a link analysis system or algorithms [47]. We used the following search terms for each website and database analyzed: “drugs and internet,” “drug/s online,” “online pharmacy/ies,” and “internet pharmacy/ies.” We scanned the reference lists for relevant articles up to the second level, and we considered the “related articles” of relevant ones in the PubMed database.

**Figure 1:**
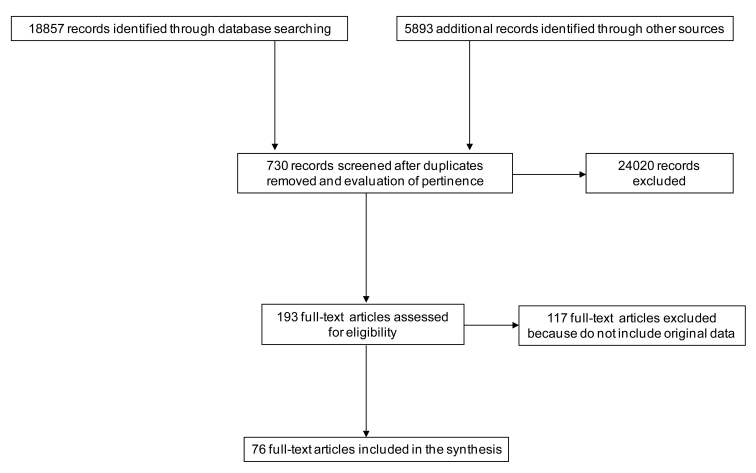
Paper selection algorithm.

The database search identified 18,857 records, and other sources (search engines and institutional websites) gave 5893 additional records. Screening of these 24,750 records led to 730 articles, excluding duplicates and nonpertinent results. It is important to point out that such a drop in numbers depends mainly on the use of multiple key words, which are often very similar, which were used in order not to miss any pertinent studies. This resulted in a notable “noise effect,” thereby decreasing the specificity and increasing the sensitivity of our search strategy. An in-depth analysis of the 730 selected articles produced 193 eligible ones that were pertinent to the study and fit the inclusion criteria. Of these, 117 where excluded from the analysis and are listed in Appendix 1, giving a final sample of 76 full articles for study. Figure 1 shows the selection process.

### Inclusion Criteria and Coding of Contents

We included all articles relevant to the subject of the research—namely, online pharmacies, their characteristics, their products, and their consumers. We selected only articles dealing with the sale of prescription-only drugs and with websites that presented themselves as pharmacies: the purpose was to stay within the sphere of substances that are supposed to involve a doctor–patient relationship. Articles regarding only over-the-counter medicines, complementary medicines, herbal remedies, supplements, and drugs of abuse were excluded. If the researchers analyzed websites selling prescription and over-the-counter drugs, we considered websites selling prescription drugs if it was possible to identify them. We decided to deal only with prescription drugs, although over-the-counter substances can also have negative effects on people’s health, despite the no-harm claims made by their producers, as several clinical cases demonstrate [48,49]. Although examining nonprescription drugs was not an objective of this review, it should be borne in mind that some case reports showed the presence of prescription drugs even in products that did not claim to contain them [50,51]. This means that prescription-only drugs can be distributed through channels in which the active substances do not appear.

As an additional inclusion criterion, we selected articles in English that had the abstract or the full text available. We included only scientific articles, which means that we excluded popular articles published in daily newspapers, and in weekly and monthly magazines.

We classified the articles according to whether they reported original data. We selected only the articles reporting original data, which means that we excluded articles lacking original data, which means those with only a speculative discussion about the problem or only citing data from other studies; these are, for example, editorials, letters, comments, articles about regulation issues, and reviews. However, to make this debate easily available to the reader, we have listed all articles without original data in Multimedia Appendix 1. A discussion of all the reviews we found is included in the introduction.

The original data are described according to 3 main subjects: types and characteristics of online pharmacies, drugs purchased online, and online pharmacy consumer data, which included case reports on complications occurring in consumers of drugs purchased online. Some articles with original data covered more than 1 of these subjects and were consequently allocated to more than 1 group. Each of these categories is described below.

#### Types and Characteristics of Online Pharmacies

If they were available, we recorded the number of online pharmacies analyzed in each study, year of data collection, willingness to dispense pharmaceuticals with or without a prescription, availability of a physician’s assistance or online medical consultation, disclosure of contact details, geographical location, delivery conditions, types of medicines available, availability of drug information, prices of online drugs and overall costs, sales-promotion strategies, how long websites were accessible, privacy and disclaimer statements, date of last website update, and presence of quality certifications (for instance, Verified Internet Pharmacy Practice Sites [VIPPS] by the NABP; the Health on the Net Foundation [HON] code; and the Joint Commission on Accreditation of Healthcare Organizations [JCAHCO]).

#### Quality of Drugs Purchased Online

We recorded studies in which the researchers bought prescription drugs online and evaluated the actual purchase and its characteristics. We summarized data regarding the type of drug ordered, the response rate, the quality of the process, and the drugs purchased. Regarding process characteristics, we recorded prescription requirements, management of the online questionnaire, money transactions, and subsequent advertising; with regard to drug quality, we recorded data about packaging and instructions, and chemical composition.

#### Consumers Buying From Online Pharmacies

We described articles dealing with the number of people purchasing drugs online, which was estimated by means of questionnaires or interviews. Researchers attempted to list the most frequently requested drugs, the main reasons for buying pharmaceutical products online, the importance of the location of online pharmacies they bought from, and the perceived risks related to this practice. In addition, we classified in this section articles that reported clinical cases of adverse effects to active substances and drugs purchased via the Internet as an indicator of this phenomenon.

## Results

We selected 193 relevant articles: 76 articles with original data (39%), and 117 articles without original data (editorials, regulation articles, or the like) including 5 reviews. Articles with original data concerned samples of online pharmacies in 47 cases, online drug purchase in 13, consumer characteristics in 15, and case reports of adverse effects of online drugs in 12.

### Types and Characteristics of Online Pharmacies 

We selected 47 articles about online pharmacies. All of them are shown in Table 1, except 5 that had no data in addition to the number of online pharmacies found (3 articles), or did not clearly discuss the theme of selling drugs on the Internet (2 articles). The first 3 articles are by Schifano et al [52], who found in the Psyconaut 2002 EU Project 165 websites offering the possibility to purchase drug-related items, Schepis et al [53], who assessed the availability of stimulants over the Internet as a function of specific search terms used in the search engine, and Lott and Kovarik [54], who assessed the availability of the dermatological medications isotretinoin and terbinafine over the Internet from illicit commercial sites. We do not show in Table 1 the other 2 articles, which focus on the assessment of community pharmacy websites in Turkey and Switzerland [55,56]. The former made only a passing reference to the presence of e-commerce services, but it was not possible to determine whether they were actually selling prescription drugs; the latter also assessed the presence of e-commerce services without referring to what was actually sold online.

**Table 1:** Contents of articles about online pharmacies, listed in alphabetical order according to first author; the presence of each item is indicated when studied and the percentage is reported when comparable; “X” indicates that the item was analyzed but could not be tabled

First author, year of publication	Prescription requirement (%)	Online questionnaire (%)	Contact details (%)	Geographical location (%)	Delivery	Drugs offered	Drug information (%)	Prices	Marketing strategies	Quality	How long websites were accessible	Privacy policy
Armstrong, 1999 [57]	0	50		100		X^a^	55	X	X		X	X
Arruanda, 2004 [58]	30		99		X	X	35	X		X	X	
Bate, 2010 [59]					X	X^a^		X				
Bessel, 2002 [60]	81	12	66	79	X	X			X	X		X
Bloom, 1999 [61]	X^b^	X^b^		11		X		X				
Bloom, 2006 [62]	X^b^	X^b^		92		X		X				X
CASA^c^, 2008 [63]	15	41			X	X^a^					X	
Cicero, 2008 [64]						X^a^		X				
European Alliance, 2008 [65]	10	16	42	16					X	X		X
Eysenbach, 1999 [66]	9	50		X		X^a^		X	X			X
Forman, 2003 [67]	0^a^			X		X^a^						
Forman, 2006 [68]	0^a^			X		X^a^						
Forman, 2006 [69]	0^a^	50				X^a^						
Forman, 2006 [70]	0^a^	52				X^a^			X	X		
Gallagher, 2010 [71]	7	59	100	59								
GAO^d^, 2000 [72]	58	28	81				61					X
GAO^d^, 2004 [73]	34	40		X		X						
Gernburd, 2007 [74]	0						100	X	X		X	
Gurau, 2005 [75]	34	59	100						X			X
Holmes, 2005 [76]							X^e^					
Koong, 2005 [14]						X						
Kunz, 2010 [77]										X		
Kuzma, 2011 [78]										X		
Levaggi, 2009 [79]	19	67		44	X	X		X	X			
Littlejohn, 2005 [80]	10	X				X		X				
Mahé, 2009 [81]	X					X^a^		X	X			
Mainous, 2009 [82]	0^a^	64			X	X^a^						
Makinen, 2005 [27]	X	X		X^a^	X	X				X		
Memmel, 2006 [83]	0	75		X		X^a^		X				
NABP^f^, 2010 [10]	4	58		47		X				X		
Orizio, 2009 [84]	19	56		43	X	X	X		X			
Orizio, 2009 [85]	0^a^	100^a^		28								
Orizio, 2010 [86]	22	45				X	X		X	X	X	
Peterson, 2001 [87]	88		67	X^a^		X	X			X		X
Peterson, 2003 [88]				X^a^		X				X	X	X
Quon, 2005 [89]				X^a^		X		X				
Raine, 2009 [90]	17	41		43	X^a^	X^a^	X					
Schifano, 2006 [91]	0	10				X^a^						
Soares Gondim, 2007 [92]			81	X^a^			X			X		
Tsai, 2002 [93]	0^a^	81	37	98		X^a^	X	X		X		
Veronin, 2007 [94]				X^a^								
Wagner, 2001 [95]	100		100	X^a^	X	X^a^	100	X				

^a^ See other specific inclusion criteria, fourth and fifth column in Multimedia Appendix 2, which lists the characteristics of the studied samples of online pharmacies.

^b^ Does not specify whether the prescription is an original one from the customer’s physician or an online one.

^c^ National Center on Addiction and Substance Abuse.

^d^ US Government Accountability Office.

^e^ The study aimed to evaluate the responses provided by the “ask the pharmacy” service.

^f^ National Association of Boards of Pharmacy.

The remaining 42 articles [10,14,27,57-95] dealing with online pharmacy characteristics are listed in Table 1, which shows the first author, year of publication, and the main features studied; due to the descriptive nature of the data it was not possible to report numerically all the results presented in the following subsections. When the numeric percentages of presence of the item were available, they are given in the table; the letter X means it was not possible to include data in the table or compare them (when, for instance, the groups they refer to are different and hence not comparable). The kind of information represented by the X is always described in detail in the text. Appendix 2 gives the sample size and the methods of selection regarding these 42 articles.

**Figure 2:**
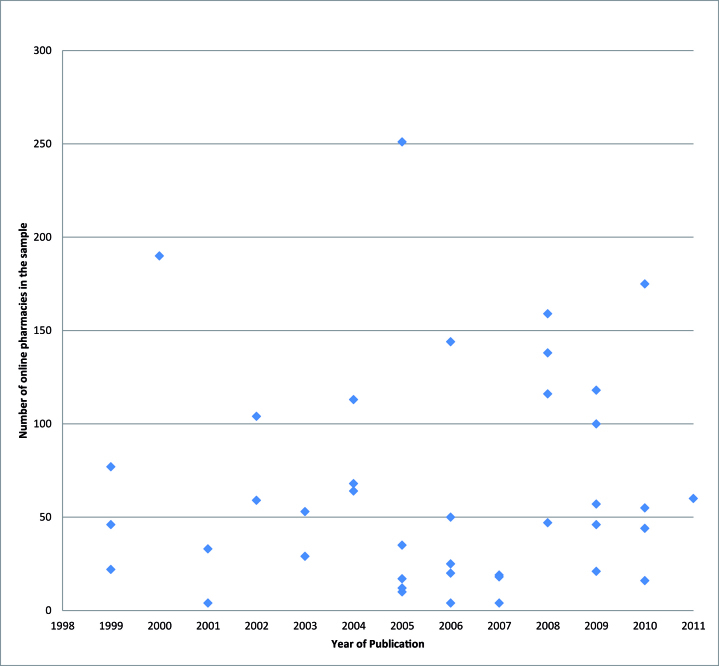
Articles on online pharmacy characteristics (included in Table 1) by year of publication and number of online pharmacies in the study sample (data not reported because out of range: NABP sample of 5859 online pharmacies [10]).

The works were published between 1999 [57,61,66] and 2011 [78], as shown in Figure 2, which displays the articles described in this section by year of publication and number of online pharmacies in the study sample. Less than half of the studies analyzed had more than 50 online pharmacies in their samples. Samples were obtained using a variety of methods, the most frequent being to look for online pharmacies using online search engines via various keywords. Some researchers used different sample selection methods: one found websites from received spam [74], while others looked for the websites indicated by other sources, such as the first one listed in the Best Online Pharmacy guide by epharmacyfinder.com [14], or the VIPPS list [59,76], the Pharmacychecker.com list [89], or the Top 100 Retailer [77].

Online pharmacy samples varied hugely in size, from 4 [83,94,95] to 5859 [10]. The sample selection had different inclusion criteria: many studies focused on online pharmacies selling numerous kinds of drugs, while others selected online pharmacies offering a single medication or 1 class; some selected only online pharmacies not asking for a prescription, and others only online pharmacies based in specific countries. These particular inclusion criteria are shown in Multimedia Appendix 2 and are described in the subsection dealing with each issue.

#### Prescription Requirement

One of the most controversial and widely studied features is prescription requirement. For the first time, online pharmacies are providing easy access to traditionally controlled (according to each country’s regulations) substances, such as pharmaceutical products, which in regulated systems need an original medical prescription before they can be bought. The peculiar distribution chain for prescription drugs is due to the unique nature of pharmaceutical products; the philosophy behind prescription is that consumers are not skilled enough to make their own choice, but need to be given a prescription by a health professional trained to make a risk–benefit evaluation [2,24]. In addition, the medical follow-up of a drug treatment is crucial. Furthermore, there are problems regarding strict quality control for substances that can determine life or death. Last but not least, there are economic implications in ruled systems where drug prices are negotiated and partially funded by the health system [27].

Due to the different methods of sample selection, distribution varies greatly in the articles. Some online pharmacy samples were specifically selected to have only websites not asking for a prescription from the customer’s physician [57,67-70, 82,93]. In the samples that did not use prescription as a criterion for inclusion or exclusion, the prescription requirement varied widely: all websites asked for an original medical prescription in Wagner et al [95] (who selected only US-based online pharmacies), more than half did so in other samples [60,72,87], less than half [10,58,63,65,66,71,73,75,80,84,86,90] in still others, and none in a few [74,83,91]. In Bloom and Iannacone [61,62] it was not possible to determine whether the required prescription was an original one by the customer’s physician or replaced by an online health evaluation as explained below.

#### Presence of an Online Questionnaire

Some of the online pharmacies not asking for a prescription replaced it with an online health status evaluation, performed by means of an “online questionnaire.” Researchers reported a percentage of online pharmacies offering and/or requiring an online prescription in a range of 10% to 81% [91,93]. Those who looked deeper into this issue found that the identity of the professional who made the prescription was not usually provided [57], whereas they often declared that the questionnaire reviewer was a physician [57,61,62,66,84] or, more rarely, a pharmacist [27,84]. Gallagher and Chapman [71] reported that, of the 26 websites using a questionnaire, 1 involved multiple-choice check-box answers and drop-down menu answers, and the others used questionnaires that allowed users to type in an answer. Eysenbach [66]—who attempted to buy the products—found that the identity of the physician who made out the prescription was revealed in 2 out of 10 orders. The US Government Accountability Office (GAO) [72,73] found the collaboration of licensed physicians, although when they verified these licenses [72] almost all of them were nonexistent or not valid for all the US states where they were declared to be. No study found the use of a validated or standardized questionnaire. Orizio et al [85] did a subanalysis of a previous study [84] that focused on the characteristics of online questionnaires; it reported that the questionnaire was already filled in with negative answers in 70% of online pharmacies using this tool, and that in only 53% of cases were different questions asked for different products ordered. Since the questionnaires were often incomplete, the authors concluded that they appeared to aim more at giving the consumer a false sense of health assurance than at actually assessing health status. The questionnaire frequently investigated personal characteristics, allergies, medical conditions, current therapies, and medical history [57,61,62,66,72,73,85].

#### Contact Details

Contact details were revealed in the majority (ranging from 100% [71,75,95], or a little less among online pharmacies when this item was analyzed [58,60,72,75,87,92,95], to 66% [60]). Only Tsai et al [93] found just 37% of pharmacies giving a telephone number for costumers’ enquiries, and the European Alliance for Access to Safe Medicines’ [65] analysis found that 42% provided a working telephone number. Bessel et al [60] pointed out that only 35% of their sample published the owners’ or the director’s names, and European Alliance [65] found that 94% of the sample did not have a named verifiable pharmacist to answer questions.

#### Geographical Location

Declaration of geographical location is an important feature with regard to transparency. Apart from the studies that focused only on websites based in a specific area, the sampled websites declared a geographical location ranging from 11% to 100% [47,61]; according to the results of Armstrong et al [57], Bessel et al [60], Bloom and Iannacone [62], Gallagher and Chapman [71], and Tsai et al [93], more than half of the samples mentioned where the company was based; the others found that less than half did [10,61,65,84,90]. Orizio et al [84] found that online pharmacies asking for a prescription were significantly more likely to declare their geographical location than were online pharmacies that did not, which were more often just virtual interfaces. Online pharmacies were most frequently based in the United States in virtually all the studies that investigated samples that were not restricted geographically [10,57,60,61,62,66,73,84]. Other studies focused on online pharmacies based in specific countries, such as the United States [87,88,95], the United States and Europe [27], the United States and Canada [89], Canada [94], or Brazil [92]. Some researchers looked for where the websites were registered, and they found the United States to be the most frequent location of domain registration [10,67,68,84,93]. Orizio et al [84], who compared the declared physical location with the registration domain, found that in only 55% of online pharmacies declaring their physical location did it correspond to the area of domain registration. Armstrong et al [57] found that US-based websites were more likely to ask for medical information and provide information about risk of treatment or its efficacy, and the GAO [73] found a better quality in websites based in the United States.

#### Delivery: Where From and Where To

The National Center on Addiction and Substance Abuse (CASA) [63] found that 24% of shipments were declared to be from the United States, and 36% of the sample gave no indications. In Orizio et al [84] 42% of the websites declared where they deliver from, the most frequent place being Asia (47%); agreement between declared physical location and delivery location was found in 31% of online pharmacies providing both details. The attempt by Bate and Hess [59] to purchase drugs showed several cases of shipments from a different location from what was indicated on the website.

Both Arruanda [58] and Bessel et al [60] found that two-thirds of the online pharmacy samples sold internationally, whereas all the websites sampled by Schifano et al [91] did. Raine et al [90] selected only online pharmacies delivering to the United Kingdom. Makinen et al [27] related the fact of shipping abroad to the prescription requirements: online pharmacies that asked for a prescription were more likely than those that did not to sell only in the country they were based in, and delivered anywhere in the world. Mainous et al [82] found that almost all the websites sold to the United States, followed by the United Kingdom (84%) and Canada (80%). None of the small sample of 4 US-based online pharmacies analyzed by Wagner et al [95] delivered internationally.

#### Drugs Offered by Online Pharmacies

What do online pharmacies have to offer? From an analysis of the literature it appears that online pharmacies have become more and more complex as time passes; whereas 10 years ago they tended to sell principally lifestyle drugs such as sildenafil, it looks today as if they offer virtually anything. Back in 1999, Bloom and Iannacone [61] studied a sample of online pharmacies in which the majority sold 1 or 2 drugs only, one for erectile dysfunction and the other for alopecia; in the same year Armstrong et al [57] found an entire sample of 77 websites selling sildenafil. In 2003 Arruanda [58] found that their sample was equally divided into 3 groups: selling 1 drug only, between 2 and 29 drugs (average 7), and selling 30 or more. The various studies reported several specific drug offers: sildenafil [59,60,80,84,86,87], benzodiazepines [84,86,87], painkillers [80,84,88], antibiotics [87,88], insulin [87,88], female hormones [87,88], antidepressants [80,84,86], alopecia medications [14,27,80], and obesity medications [59,80]. Bloom and Iannacone [62] reported that 160 separate medications were offered in their sample, which gives an idea of how widely differentiated the drug offer is. Quon et al [89] compared the offers of 12 Canadian-based Internet pharmacies with 3 US-based drug chain pharmacies for 44 different drugs. The GAO [73] found that some drugs were more widely available and easier to purchase (Celebrex, Lipitor, Viagra, and Zoloft) than others, which were available from fewer sources or were more difficult to obtain (Accutane and Clozaril). As we will see in more detail in the next section on actual purchase, what is hard to obtain—despite appearing to be available—is US class II and III opioid analgesics, as found in Peterson and colleagues’ studies in 2001 [87] and 2003 [88] and confirmed by the GAO 2004 [73]. In the United States, “Under the Controlled Substances Act all substances that are regulated under existing federal law are placed in one of five schedules on the basis of the substances’ medicinal value, harmfulness, and potential for abuse or addiction. Schedule I is reserved for the most dangerous drugs that have no recognized medical use, while Schedule V is the classification used for the least dangerous drugs.” [72]. The NABP [10] survey showed that 14% of websites dispensed controlled substances as defined by the Act cited above, and 40% foreign or non-FDA-approved drugs.

Some studies focused on specific classes of drugs. They looked for Parkinson disease medications [95], 3 types of controlled substances (opioids, and central nervous system depressants and stimulants) [63], opiates [64,67-70]), analgesics [90], dextropropoxyphene (a painkiller) [91], antibiotics [82], and specifically ciprofloxacin [93], contraceptives [83], the erectile dysfunction medication sildenafil [57,66,71], and psoriasis medications [81].

Littlejohn et al [80] and Makinen et al [27] linked the drugs offered to the type of online pharmacy: “legitimate pharmacies” did not supply opioids or ritalin [80], selling only over-the- counter products and herbal, hygiene, and cosmetic products [27]; “lifestyle pharmacies” supplied erectile dysfunction and alopecia medications; and “no-prescription pharmacies” supplied virtually everything, including opioids [80] and unapproved pharmaceuticals [27]. Both generic and brand drugs were available on the market [14,58,79,89,95].

#### Presence of Information About the Drugs for Sale

In 1999 Armstrong et al [57] found that 55% of their sample included drug information. Several researchers who attempted to evaluate the presence of information on side effects found that a fairly consistent portion of online pharmacies, ranging from a quarter to a third, declared none of them [84,86,90,92,93]. Arruanda [58] found that 35% of his sample provided a service allowing buyers to consult experts about the use of medicine. In the GAO [72] sample, 61% of websites gave drug information. Wagner et al [95] found in all the online pharmacies in their small sample (4 US-based online pharmacies) more comprehensive information than received from the community store. Peterson [87] did not find a statistically significant difference between the types of pharmacies and the provision of drug information, although it should be noted that his sample was small for performing a group comparison (33 online pharmacies). Gernburd and Jadad’s [74] research based on spam offers found that all of the websites in their sample made benefit claims and warned about potential side effects. Interestingly, an attempt to count the declared side effects of 4 drugs tracked in online pharmacies that asked for a prescription and those that did not revealed that no-prescription online pharmacies declared more side effects for amytriptiline, fluoxetine, and tramadol, but fewer for sildenafil, which—probably not by accident—is one of the most widely offered “lifestyle drugs” in online pharmacies [84,86].

Holmes et al [76] evaluated the quality of online pharmacy “ask the pharmacy” services regarding adverse effects, drug interaction, risk factors, drug information, and directions for use. They found that answers were received to only 51% of all questions submitted to the websites, and the percentage of correct answers provided for each of 22 response components ranged from 7% to 96%, with few differences in quality between VIPPS-approved and -unapproved websites.

#### Prices

Arruanda [58] evaluated the presence of drug price lists (found in 96% of the sample) and of “price comparisons with competitive pharmacies” (1%).

A comparison of online pharmacy and retail pharmacy prices had different results. Drugs offered online were more expensive in Bloom and Iannacone’s studies [61,62], in Tsai and colleagues’ [93] analysis, and in Cicero and colleagues’ [64] purchase. Wagner et al [95], who compared prices of US retail versus US online pharmacies, found that the latter were cheaper for both generic and brand-name medications. Levaggi et al [79] found that drugs bought without a prescription cost more than with one. In Bate and Hess’s [59] purchase, larger orders generally tended to have lower per-tablet/per-capsule prices, and were more prevalent among online pharmacies that had not been approved by the NABP. Interestingly, they found that Viagra offered on noncredentialed websites was on average far more expensive than from credentialed ones, and for all the drugs purchased (Lipitor, Celebrex, Nexium, and Zoloft), except Viagra, prices were higher at physical-location pharmacies. Quon et al [89] compared US retail prices with those of Canadian online pharmacies, and they found savings when purchasing from Canadian online pharmacies for the majority of brand drugs (3 exceptions in the 44-drug sample were medications for erectile dysfunction), whereas generics were more expensive. The same result is confirmed by an aggregate macroeconomic analysis based on IMS Incorporated data on prices, patents, and cross-border Internet pharmacies between online Canada and retail US pharmacies [15]. Memmel et al [83] found lower prices for contraceptives purchased online. Mahé et al [81] found that, with the exception of tazarotene, the average price of all the online psoriasis medicines analyzed was higher than the French retail price.

Additional costs have to be considered: the need to join clubs or member groups and pay a nonrefundable fee, with the risk of not finding the wanted drug after joining [64,80]; and delivery costs, with free delivery in some online pharmacies or for some purchases, but with charges depending on the type of shipping in other cases (standard, express, overnight) [57,58,61,62,66, 74,79,89,95]. Online prescription could be another added cost [57]. It is interesting to note that, although a prescription is not necessary in marketing messages, Levaggi et al [79] found in their price analysis that the prescription has a value on the market; indeed, when you buy a drug without a prescription, the drug costs more.

#### Marketing Strategies

Only a few articles focused on the marketing strategies of online pharmacies. Levaggi et al [79] and Orizio et al [84] disclosed the persuasive statements more frequently used by websites to promote their products, and identified arguments regarding privacy, service and drug quality, price offers, reassurances that buying drugs online is legal, and the suggestion that you can obtain a drug while avoiding a visit to the doctor. In particular, privacy issues were about the use of personal data and discreet packaging; service quality statements regarded short delivery times, online tracking of the state of the orders, and—indirectly—displaying testimonials by people who had already bought online; and price offers referred to encouragement to buy bulk purchases (found by Armstrong et al [57], European Alliance for Access to Safe Medicines [65], and Mahé et al [81] as well), lower prices than in “brick and mortar” pharmacies, fidelity bonuses, free delivery, and special discounts [79]. Armstrong et al [57] reported that one-third of their sample pointed out the advantages of ordering online, including confidentiality, ease of ordering, and lower cost. Forman and Block [70] analyzed the implied legitimacy and credibility claims, which they divided into 3 types: medical legitimacy, found in 82% of online pharmacies (eg, pictures of lab coats, rx/health symbols, and pharmaceutical logos); legal legitimacy, found in 72% (eg, government logos, explicit and implicit claims of being legal, and FDA approval statements); and retailer legitimacy, found in 24% (eg, customer testimonials). They also reported claims about shipping regarding security (52%), secretiveness (70%), reshipment if seized (2%), risk of seizure by US customs (6%), free delivery (24%), and the delivery company (52%). All the websites in Gernburd and Jadad’s [74] sample put forward benefit claims. One website in Eysenbach’s [66] sample even offered to ship cimetidine together with a Viagra order because it causes “a 56% increase in plasma sildenafil concentration when co-administered”. Gurau [75] pointed out that providing contact information may be a way to reduce the perceived risk of online transactions; moreover, statements about price, convenience, choice, and discreetness of service are more frequently used by online pharmacies not asking for a prescription than by those asking for one. Advertising prescription-only medicines was also found by Bessel et al [60] in 20% of online pharmacies. Orizio et al [86] pointed out that the marketing strategies adopted by online pharmacies enhance consumers’ peripheral reflection: by analogically playing with the sale of other commodities, they magnify aspects of online trading that consumers might find convenient, but overshadow the nature and risks of the actual products they sell.

#### Quality

The presence of at least 1 quality certification was found to range from 12% to 13% [60,70,86] to 28% [58], but Tsai et al [93] did not find any websites displaying certificates. Researchers found certificates about the quality of the health contents and/or about security in the money transaction.

Regarding health content quality, Arruanda [58] found that the most frequent quality certificate was VIPPS [96] (9%), followed by the HON code [97] (5%). Bessel and colleagues’ [60] sample had quality certification on 12% of the websites: the national pharmacy authority, the HON, and the JCAHCO [98]. Makinen et al [27] reported finding the HON code and VIPPS seal. Peterson’s sample in 2001 [87] had 12% of websites with the VIPPS seal, rising to 18% in 2003 [88]. The European Alliance for Access to Safe Medicines [65] study found that 4% of the sample was licensed by a board of pharmacy or had an appropriate pharmacy listing, while 20% had a “stamp approval” from a recognized society or association, but they found when clicking on them that 86% gave a link to a bogus “approval” webpage. Soares Gondim and Borges Falcao [92] found that 15 of the 16 websites analyzed lacked the Brazilian National Health Surveillance Agency seal.

As regards the security of the money transaction, Arruanda [58] found the VeriSign seal [99] (8%), Forman and Block [70] found Verified by Visa and Master Card securicode logos, and Makinen et al [27] reported finding the TRUSTe seal [100]. As well, 24% of Peterson’s sample in 2001 [87] incorporated secure socket layer technology, rising to 33% in 2003 [88].

A special study was performed by Kuzma [78] about the Web vulnerability of a random sample of 60 online pharmacies. She chose as her testing tool the N-Stalker Web Application Security Scanner 2009 Free Edition 7.0, which showed that a majority of worldwide online pharmacies do not provide adequate protection for their consumers, especially in cross-site scripting. The NABP [10] found that 17% of the websites in their sample did not have a secure site.

Kunz and Osborne [77] assessed the readability of 16 online pharmacies using Storytoolz; they found that the majority of the information provided on direct-to-consumer pharmaceutical websites is written at a level far higher than that which the average consumer can understand.

#### Website Time of Existence

Arruanda [58] found that the sample online pharmacies had been online for a length of time ranging from just over 1 year to 7 years. Armstrong et al [57] reported that in the 10 days between website identification and data collection 9 (12%) ceased operating. In the CASA study [63] only 2% of the 152 non-VIPPS anchor sites identified in 2004 were still operating in 2008. When Orizio et al [86] rechecked in 2008 the 118 websites found in 2007, 75% were working. The follow-up of Peterson’s [87] study found that 1 year later 88% of the initial 33 online pharmacies were still working [88]. Gernburd and Jadad [74], who worked on links addressed by advertising spam, found that only 58% of the active link in health-related messages received during the first week of the study remained active at the end of the second week, whereas 26% were active at the end of the month.

#### Privacy Policy and Disclaimers

In several of the papers analyzed we found that some websites required the consumer to relieve the companies from all liability, from 100% of websites [62,66], to 68% [57], 50% [65], and 33% [72]. Bessel et al [60] found privacy statements, information disclaimers, and return policies on 40%, 31%, and 37% of the websites, respectively, while 37% of websites displayed none of these policies. The GAO [72] found privacy statements on 23% of the sample. Gurau [75] found that websites displayed their privacy policy more frequently when they asked for a prescription (98%) than if they asked for an online questionnaire to be filled out (86%) or asked for nothing (56%). Peterson [87] found that a privacy policy was present in a significantly different way in the various types of pharmacies that he identified (chain extension 100%, mail order 16%, online 64%, independent extensions 25%); the same author reported in 2003 [88] that several websites had added a privacy policy.

#### Last Website Update

Only 1 study investigated the date of the online pharmacies’ last update, which was displayed by 4% of them (data not shown in Table 1) [60].

### Drugs Purchased Online: Process Characteristics and Drug Quality

We found 13 studies [59,64-66,73,74,82,83,94,101-104] in which the researchers bought prescription drugs online and evaluated the actual dispatch and its characteristics, and the quality of the products received (Table 2). The response rate ranged from 30% [66] or over (39%) [74] to almost 100% [65,101] or 100% [59,73,74,102], except for the research by Cicero et al [64], who received nothing after ordering opioids, whereas his only order for tramadol was successful. It should be noted that the various groups of researchers ordered and purchased different types of drugs from different samples of online pharmacies. Some were interested in specific categories of drugs [64,66,82,83,94,101,102,103], whereas others ordered several active ingredients [59,65,73,74,104]. Cicero and colleagues’ attempts to buy class II and III opioid analgesics did not result in their dispatch [64], but hormonal contraceptives and simvastatin were always delivered when ordered [83,94], despite a much lower number of total orders. The GAO [73], which received 75% of orders, showed the same result, as they found that top-selling drugs such as Celebrex, Lipitor, Viagra, and Zoloft were readily available from multiple Internet pharmacies, whereas other drugs, such as those with special safety restrictions (Accutane and Clozaril) and narcotic painkillers (Percocet, OxyContin, and Vicodin), were offered for sale by fewer Internet pharmacies or were otherwise more difficult to obtain.

**Table 2:** Articles on the quality of drugs purchased online, listed in alphabetical order according to the first author

First author, year of publication	Year(s) of data collection	Type of drugs ordered	Response rate (products received/number of orders)	Drug purchase characteristics	Drug quality characteristics
Bate, 2010 [59]	2009	Lipitor, Viagra, Celebrex, Nexium, Zoloft	Response rate not computable; 152 ordered drugs were received	Prescription requirement, money transaction	Packaging, chemical analysis
Cicero, 2008 [64]	2006	Opioid analgesics	0% (0/47) of “opioid scheduled” orders, 100 (1/1) purchase of tramadol	Prescription requirement, money transaction, subsequent advertisement	
Dean, 2010 [102]	Not declared	Dapoxetine	100% (1/1)		Chemical analysis
European Alliance, 2008 [65]	Not declared	18 different active ingredients^a^	94% (34/36)	Prescription requirement	Packaging, instructions, chemical analysis
Eysenbach, 1999 [66]	1999	Viagra	30% (3/10)^b^	Prescription requirement, management of online questionnaire, money transaction	
GAO^c^, 2004 [73]	2004	13 different active ingredients^d^	75% (68/90)	Prescription requirement, money transaction	Packaging, instructions, chemical analysis
Gernburd, 2007 [74]	2006	13 different active ingredients^e^	39% 5/13^b^	Prescription requirement, money transaction	
Mainous, 2009 [82]	2008	Antibiotics	100% (1/1)	Prescription requirement	Instructions
Memmel, 2006 [83]	2004–2005	Hormonal contraceptives	100% (10/10)^f^	Prescription requirement, management of the online questionnaire	Packaging, instructions
Miller, 2001 [101]	1999–2000	Prescription and nonprescription contraceptives	96% (9/10 in 1999 and 15/15 in 2000)	Prescription requirement, money transaction	
Veronin, 2004 [103]	Not declared	Simvastatin	Response rate not computable; 5 ordered samples were received		Chemical analysis
Veronin, 2007 [94]	2006	Simvastatin	100% (4/4)^g^		Chemical analysis
Westenberger, 2005 [104]	Not declared	Fluoxetine, levothyroxine sodium, metformin hydrochloride, phenytoin sodium, warfarin sodium	Response rate not computable; 20 ordered samples were received		Packaging, instructions, chemical analysis

^a^ The drugs purchased were Cialis, Levitra, Viagra, Propecia, Lipitor, Plavix, Seretide, Coversyl, Micardis, Spiriva, Zyprexa, Efexor, Risperdal, Aricept, Reminyl, Zoton, Reductil, and Mirapex.

^b^ Orders made only on websites asking for an “online questionnaire” to be filled in to obtain an online prescription. The online questionnaire was completed by a fictitious patient with clear contraindications for sildenafil.

^c^ US Government Accountability Office.

^d^ The drugs purchased were Accutane, Celebrex, Clorazil, Combivir, Crixivan, Epogen, Humulin N, Lipitor, and OxyContin.

^e^ The drugs purchased were Ambien, Celebrex, Cialis, Meridia, Nexium, Propecia, Soma, tramadol, Valium, Viagra, Xanax, Zithromax, and Zoloft.

^f^ Orders made with different risk profiles.

^g^ Orders from Canadian websites only.

#### Drug Purchase Characteristics

The actual purchase of drugs without having an original medical prescription was verified by all authors except for Dean et al [102], Veronin and Youan [103], Veronin et al [94], and Westenberger et al [104], who did not specify whether a prescription was used. Two studies evaluated the effectiveness of online questionnaires for assessing health status. The results highlighted the low performance of this tool, which allowed the purchase of contraindicated products by fictitious consumers in some or all orders [66,83].

Several authors found a lack of reliability in the business practices of some online pharmacies, linked to completed money transactions without actual receipt of the drugs [66,73,74,101] or having to join clubs, only to find no drugs available after having paid the necessary fee [64]. One study reported subsequent advertising by email and phone for more than 4 months after ordering [64].

#### Drug Quality Characteristics

Regarding drug quality characteristics, researchers evaluated different features: the packaging of the drugs purchased, the instructions included, and the chemical composition.

As to packaging characteristics, packaging showed problems in more than half of the drug samples in the GAO [73] study. Memmel et al [83] found that all packaging appeared legitimate and products were within the expiration date, but patient education material (instructions) did not always match the product. GAO [73] researchers found better quality instructions in samples from US and Canadian Internet pharmacies, which in all 47 samples included dispensing pharmacy labels that generally provided patient instruction for use and in 87% of cases included warning information, compared with other foreign pharmacies, whose labeling and facility of manufacture were not FDA approved in the majority (19/21) of cases. Westenberger et al [104] found that only 1 of 20 samples had final packaging, including package insert, similar to that of the US products. The final packaging of the remainder consisted of bubble wrap inside a paper envelope, a Styrofoam sheet inside a paper envelope, loose blister packs, capsules or tablets in clear plastic bags without labels, and capsules or tablets in opaque plastic containers or boxes with labels. Bate and Hess [59] reported that many drugs, including some that did not fail in spectrometry testing, had an unclear or problematic origin or problematic packaging. In the European Alliance for Access to Safe Medicines study [65], 47% of the drugs purchased had no packaging, and in 6% the packaging had been tampered with; half of the sample supplied a patient leaflet, which was in English in 80% of cases. Mainous and colleagues’ [82] purchase arrived with no instructions.

The GAO [73] found that the chemical composition of 4 samples out of 68 was not comparable with the product ordered: in 2 cases a counterfeit version contained less active ingredients, and in 2, a significantly different chemical composition from the product ordered. An analysis of counterfeit dapoxetine (a short-acting selective serotonin-reuptake inhibitor), used against premature ejaculation, found that the tablets contained undisclosed sildenafil [102]. Bate and Hess’s [59] analysis showed that 2.5% (3/121) of the tablet sample failed Raman spectrometry. Westenberger et al [104] found that 2 out of 20 samples failed in terms of dissolution and chromatographic purity; Veronin and Youan in 2004 [103] found differences in simvastatin tablet formulation, obtained from 5 countries via the Internet, using near-infrared spectroscopy chemical imaging methods to assess blend uniformity. In contrast, Veronin et al in 2007 [94] found a quality standard comparable with that of the American manufacturer and the Canadian generic drug product tested. The European Alliance for Access to Safe Medicines study [65] study found that 62% of the products received were counterfeit, substandard, or unapproved medications (68% of these were generic and 32% were branded).

### Consumers Purchasing From Online Pharmacies

The most difficult task in connection with online pharmacies is attempting to establish the number of people buying and the volume of money traded. Except for cases when this practice is a legal requirement, there are no official data on the issue and it was not possible to obtain details from the Internet as in the sections above.

The scientific evidence about consumers comprises 2 types of data: population surveys and case studies on the adverse effects of drugs purchased via the Internet.

#### Population Surveys

Table 3 shows the 15 articles [12,64,75,105-116] dealing with consumer characteristics and based on surveys. Most studies were US based, except for 4, which were conducted in Europe [75,105,106] and South America [107]. The findings were published between 2003 [108] and 2010 [105-107,109-112]. Seven studies investigated the general population [12,75,106,108,109,113,114], while the remainder were about specific groups, described in Table 3 [64,105,107,109,111,112,115,116].

**Table 3:** Articles about consumers buying from online pharmacies, listed in alphabetical order according to the first author

First author, year of publication	Year(s) of data collection	Country where the study was performed	Population investigated	Study design	Percentage of people buying prescription drugs online
Atkinson, 2009 [113]	2005	US	Sample of general population	HINTS 2005 survey^a^	13% (715/5586) (bought medicines or vitamins)
Baker, 2003 [108]	2001–2002	US	Sample of general population	Internet survey	5% (over 3668 respondents)
Bechara, 2010 [107]	2009	Argentina	Healthy young men	Questionnaires on use of phosphodiesterase type 5 inhibitors	2.9% (2/321)
Cicero, 2008 [64]	2006	US	Prescription drug abusers	Questionnaires	6% (41/685)
Cohen, 2010 [109]	2009	US	Sample of general population aged 18–64 years	NHI Survey 2009^b^	6% of 7192
Fox, 2004 [12]	2004	US	Sample of general population	Telephone interviews	4% (93/2200)
Gordon, 2006 [115]	2003–2004	US	Drug-dependent inpatients	Semistructured interviews	6% (6/100)
Gurau, 2005 [75]	2004	UK	Sample of general population	Semistructured questionnaires	34% (102/300) (people buying or intending to buy online)
Harte, 2010 [110]	2006–2007	US	Male college and university students	Online questionnaires on use of phosphodiesterase type 5 inhibitors	12% (8/77) of users
Inciardi, 2009 [116]	Varies with source	US	Drug abusers, students, street sex workers, and “club culture” population	RADARS System^c^, NSDUH^d^, Delaware School Study, Miami street studies, and qualitative studies	1%–6%
Inciardi, 2010 [111]	Varies with source	US	Drug abusers, students and young adults	RADARS System^c^, NSDUH^d^, MTF^e^	0.5%–3%
Mazer, 2010 [112]	2007	US	Sample of emergency department patients	Questionnaires	5.4% (89/1654)
Rajamma, 2009 [114]	Not declared	US	Sample of general population born 1946–64	Online questionnaires to a sample from the consumer panel by Common Knowledge Research Services	Not applicable^f^
Schnetzler, 2010 [105]	2008	UK, Germany, Italy	Sexually active men	Online questionnaires on use of phosphodiesterase type 5 inhibitors	32%^g^
Wiedmann, 2010 [106]	2008	Germany	Sample of general population	Face-to-face interviews	Not applicable^f^

^a^ Health Information National Trends Survey by the National Cancer Institute.

^b^ National Health Interview Survey.

^c^ Researched Abuse Diversion and Addiction-Related Surveillance System.

^d^ National Survey of Drug Use and Health.

^e^ Monitoring The Future.

^f^ Evaluation of online drug shopping attitude related to cognitive characteristics.

^g^ 32% of Viagra users obtained the drug from sources outside the health system, including the Internet.

In studies about the general population, the percentage of people buying drugs online was between 4% and 6% in the United States in the studies by Fox [12], Baker et al [108], and Cohen and Stussman [109] (who analyzed the health information technology questions of the National Health Interview Survey). Atkinson et al [113] (who analyzed the data of the US national representative sample of the Health Information National Trends Survey [HINTS] by the National Cancer Institute) found a higher percentage (13%), probably because the purchase of vitamins was included in the estimate. The UK-based survey considered both having bought and the intention to buy, so a third of the sample gave a positive response [75].

As in the general population studies, Mazer et al [112] found that 5.4% patients at an emergency department had bought drugs online.

Both surveys on prescription drug abusers [64,115] reported that 6% of the interviewees had used the Internet to purchase prescription medications for their addiction. The 2 papers by Inciardi et al [111,116] were based on several sources of information investigating different populations, including Internet-savvy high school and college students, chronic drug users, and members of the general population. Inciardi et al [116] tried to reveal the “black box” of drug diversion (the transfer of a prescription drug from a lawful to an unlawful channel of distribution or use) using several sources of information: the Researched Abuse Diversion and Addiction-Related Surveillance (RADARS) System, the National Survey of Drug Use and Health (NSDUH), the Delaware School Survey, several Miami street studies, and 2 qualitative studies. The authors concluded that, although the Internet is indeed a source for prescription drugs, the overwhelming volume of purchases is probably at the wholesale level, since few end users report accessing the Internet for drugs. In the various population groups analyzed in this study, the percentage of people accessing prescription drugs via the Internet ranges from 1% to 6%. The results of the 2010 work by Inciardi et al [111] seem to confirm the findings of the previous paper: based on the RADARS System, the NSDUH, and the Monitoring The Future (MTF) survey, they still found that the Internet is a source of prescription opioid acquisition for 0.5%–3% of the investigated populations.

A US survey on the recreational use of erectile dysfunction medications in undergraduate men showed that 12% of users had bought them on the Internet [110]. Bechara et al [107] in Argentina investigated the recreational use of PDE5Is by healthy young men and found that 2.9% of the interviewees had bought the drug through the Internet. A cross-sectional Europe-based observational study examining the purchasing patterns for the same substances found that 11% of the 11,889 subjects reported current use of PDE5Is; 32% of these reported obtaining their PDE5Is from sources outside the health care system without prior health care personnel interaction (eg, Internet, friends) [105].

Some reports suggest widespread use of online pharmacies in people over 35 years of age [75,113] and in women [109]. Mazer et al [112] found no difference in age between those who bought drugs online and those who did not, and no difference in student status, but patients on multiple medications and those with prescription plans used online pharmacies more frequently. Fox [12] found that the most frequently bought products were drugs for chronic conditions (75%), followed by weight loss and sexual performance substances (25%). The most frequent reasons quoted by interviewees for buying or intending to buy online were convenience and saving money [12,75], followed by information anonymity and choice [75]. Regarding location of online pharmacies, the majority of buyers chose or would have chosen sites based in their own countries or in economically developed countries [12,75]. Regarding risk perception, Gurau’s [75] interviewees reported being worried by a lack of a license on the part of the pharmacy (31%), privacy issues (27%), security of online payment (26%), additional charges, drug quality, and superficial prescription. It is interesting to note that health-related risks (drug quality and prescription requirement) rank last in consumers’ perception. In Fox’s [12] survey 68% agreed that online purchasing makes it too easy to obtain drugs illegally.

Rajamma and Pelton [114] explored the potential impact of consumers’ cognitive characteristics on their decision making as it relates to procuring pharmaceutical products via online retail channels. They sent 350 consumers a self-administered electronic questionnaire. Their analysis showed that male, higher-educated, and higher-income consumers had a greater propensity to procure medication online, whereas insurance status did not have any influence. Wiedmann et al [106] investigated the consumer-perceived values and risks related to online shopping attitude and behavior in an e-pharmacy context; they used their model to identify 4 clusters labeled as “enthusiastic experts” (29%), “risk-averse traditionalists” (24%), “convenience-oriented rationalists” (20%), and “inexperienced opponents” (28%).

#### Clinical Case Reports

We found 12 published papers on clinical cases related to prescription drugs obtained via the Internet, which are shown in Multimedia Appendix 3. One regarded the Internet purchase and injection of gamma-butyrolactone by an 18-year-old woman that led to admission to a pediatric intensive care unit [117]. Romero et al [118] reported florid withdrawal delirium following the discontinuation of Fioricet (a combination of butalbital, which is a barbiturate, with caffeine and acetaminophen indicated for muscle contraction headaches) that a 37-year-old woman purchased online and self-administered in escalating doses for the treatment of chronic headaches in a context of a history of depression. A 40-year-old woman was treated at the emergency department for severe tetany-like spasms, probably due to the ingestion of haloperidol and bentazepam purchased on the Internet, which were displayed on the online pharmacy under “sleep aids” [119]. Neuberg et al [120] reported a case of life-threatening thyroid hormone abuse in a 56-year-old woman encouraged and enabled by unconventional health advice and nonprescribed medication obtained via the Internet. Carisoprodol (a muscle relaxant) withdrawal after Internet purchase was reported by Eleid et al [121]. Another report described a 43-year-old woman who underwent surgery for brain cancer; after hospital discharge she researched adjunctive treatments of cancer on the Internet and self-initiated a 10-day course of cesium chloride, ending up with an acquired long QT syndrome [122]. A 55-year-old man with squamous cell carcinoma of the maxillary sinus, who declined to undergo surgery, radiation, and chemotherapy, decided to treat his cancer using hydralazine sulfate, obtained online; he died from fatal hepatorenal failure, probably caused by the hydralazine sulfate [123]. A series of case studies was reported by Lineberry and Bostwick [124] regarding a suicide attempt by a 35-year-old man using amitriptyline purchased online unbeknownst to his psychiatrist, who had prescribed him paroxetine, and 3 stories of opiate and painkiller addiction in a 37-year-old man, a 42-year-old man, and a 29-year-old woman facilitated by online purchase. Levesque [125] reported tardive dyskinesia in a 67-year-old man associated with the online purchase of the older antipsychotic drugs he probably received when he requested a tranquilizer. All the cases reported so far occurred in the US, although other evidence shows that the phenomenon exists in Europe as well. A case of prolonged hypogonadotropic hypogonadism caused by anabolic steroids purchased on the Internet by a 34-year-old Italian man was reported by Pirola et al [126]. In the United Kingdom, orlistat-induced subacute liver failure was reported in a 57-year-old woman [127]. Also in the United Kingdom, acute coronary syndrome was diagnosed in a 41-year-old man who had taken Viagra for erectile dysfunction [128].

## Discussion

We synthesized the scientific literature on online pharmacies by performing an up-to-date and comprehensive review scanning several scientific and institutional databases, with no publication time limits, focusing on the broader picture of online pharmacies. We thought it was necessary to implement a new review because we found no recent summarizing material in the scientific literature, since the second of the only 2 available reviews with a general approach cited just 14 papers regarding online pharmacy supply and was up-to-date to 2007. This is quite a considerable time in a rapidly changing world such as the Internet. In addition, 63% (48/76) of all the studies we found with original data were published in or after 2005, which seems to indicate that the phenomenon of purchasing medications online is increasing.

The main challenge in conducting this review was that the works we further analyzed and report were fairly difficult to compare owing to the widely differing methods used to select and assess samples, and often the works were written in answer to multiple research questions. In order not to miss the multiple aspects of these works we elaborated different research questions—in our opinion the only way to analyze these data. This could be seen as a limit of the study in terms of coherence, but we prefer to view it as an added value of this review, since we wanted to impress on the reader the complexity of the papers’ methods and data, mirroring the complexity of the phenomenon of online pharmacies.

**Figure 3:**
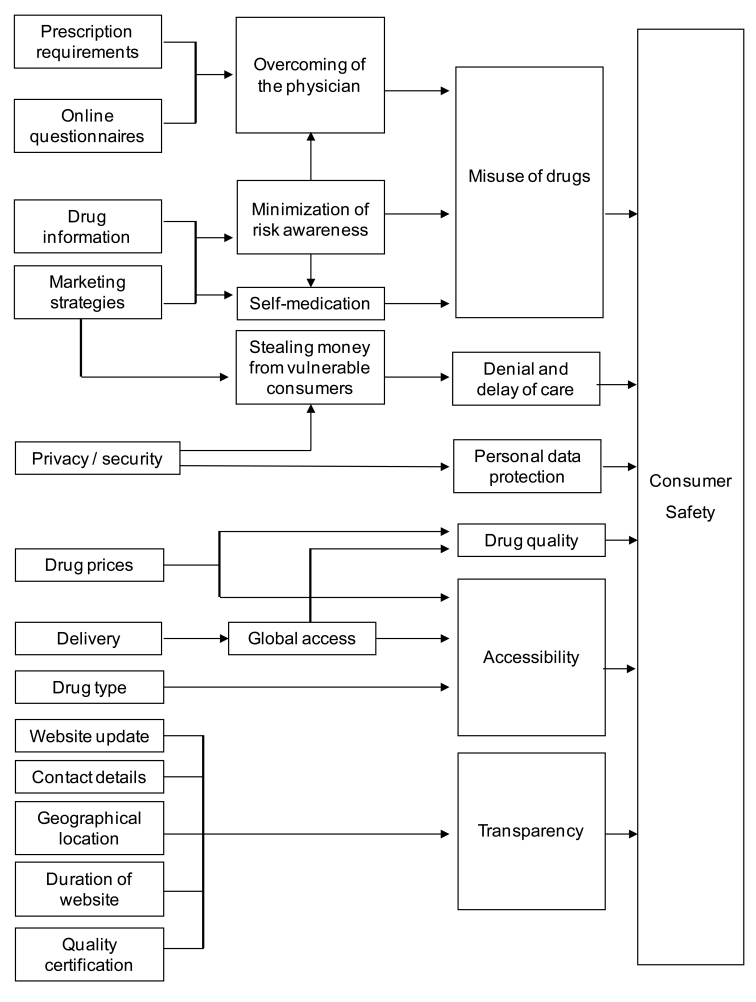
Consumer safety as a common denominator for studying online pharmacies.

Despite this complexity, we made an effort to identify a common denominator in all the research questions scholars have used in tackling the issue, and we related them back to the broad issue of consumer safety in its multiple variations. Ultimately, the tangible side of this consumer safety framework is clinical reports of health damage caused by drugs purchased online, the last link in the chain, when the feared dangers have already occurred. Consumer studies have tried to estimate the number of exposed people and to identify at-risk groups. The majority of these studies focused on the general population or specific groups, and found that 6% or less of the sample had bought drugs online. As depicted in Figure 3, all the contents that we systematized in our research can be linked to consumer safety, in terms of drug misuse, denial and delay of care, transparency issues, drug accessibility, drug quality, and consumer data protection.

One of the major risks posed by online pharmacies is drug misuse. Prescription requirement and use of online questionnaires can be linked to avoiding the physician and hence to the possible misuse of drugs. All the random samples with no specific limits regarding prescription requirements found that at least some websites sold drugs without a prescription, and that online questionnaires could be used as a substitute for prescriptions. This issue leads to the risk of one of the most feared consequences: the possible disruption of the doctor–patient relationship, which has been widely discussed. In the context of the doctor–patient relationship, drugs purchased online can have acute effects, and even chronic and irreversible ones, and nowadays doctors should always investigate the use of nonprescribed substances in their anamnesis [125]. Also connected to drug misuse is the theme of self-medication: in this context drug information displayed by online pharmacies is supposed to be a tool to help consumers be aware of the risks they are exposed to when taking a specific drug. If drug information is not available, this could minimize risk awareness. Risk awareness can also be minimized by marketing strategies, as the findings have often shown that online pharmacies tend to market their products as if they were any other commodity. Indeed, inflation of drug demand has been suggested as an effect by the papers that have analyzed aspects related to marketing strategies adopted by online pharmacies. The demand for drugs is enhanced not just by advertising economic advantages (the true nature of which appears confused and controversial when comparing works that tackled the issue), showing that there are many triggers causing a person to buy online, not just a cheaper purchase, especially in countries other than the United States where drug prices are often negotiated; the other factors probably involved are confidentiality and willingness to avoid the doctor [27,79]. Whatever the reasons, the phenomenon is likely to increase, in a context in which people are becoming increasingly accustomed to online commerce, which is increasing day by day in terms of sales volumes and the number of people engaging in it. Being more accustomed does not mean always being more aware of risks: an experimental study about risk perception in young US consumers regarding “rogue” online pharmacies showed a worrying inability to see multiple signs of danger and a tendency to be misled by online sellers that use professional design, veil untrustworthy features, and mimic reputable websites [129]. The risk of getting unnecessary drugs is also linked to pressure from marketing strategies.

Privacy issues are about the confidentiality of consumers’ data and personal data protection. The evidence suggests that online pharmacies (or ones presumed to be such) could be a tool for data fishing or fraud when they do not deliver the products (sometimes charging the consumer anyway) or send something different from what was ordered. It is not just a matter of privacy; it becomes a matter of security. This could even mean that stealing money from vulnerable consumers could lead to them not being able to afford the drug or having to wait to obtain it elsewhere, thus posing problems of denial of care and delay of care.

In terms of drug quality, when the researchers bought drugs online, they very frequently found inappropriate packaging and labeling, whereas the chemical composition was not as expected in a minority of the samples of studies, except for one [65].

Drug accessibility is a core issue regarding online pharmacies. Worldwide delivery eliminates national barriers for consumers; but the place of dispatch can be indicative of the place of production, and therefore it could be linked to drug quality. The analysis of the drugs on offer showed that an online consumer can get virtually anything, which is a matter of risk as well as accessibility, since some drugs are more intrinsically dangerous than others. Lastly, prices can modify drug accessibility and could be linked to drug quality.

Another important area associated with consumer safety is transparency in giving consumers details of the company they are buying from; this aspect can be assessed by analyzing the contact details, geographical location, time websites were accessible, quality certifications, and last website update. Geographical characteristics showed that this information is concealed on many websites, and that US-based websites tend to behave better than others. Studies that investigated the presence of quality certification found it in a minority of the websites.

### Conclusions

From a policy point of view, online pharmacies are only partially regulated due to intrinsic difficulties linked to the impalpable and evanescent nature of the Web and its global dimension, with no national barriers. The legal implications are really challenging, since the virtual “brave new world” created by the Internet poses issues never faced before. The fragmentary picture of online drug trading regulations is a recognized issue, and a noteworthy attempt to regulate the phenomenon is the “Implementation of the Ryan Haight Online Pharmacy Consumer Protection Act” by the US Department of Justice in 2009 [130-132]. Liang and Mackey [133] proposed a statute that includes drug access costs, prohibition of Internet sales, a legal reform to give several federal agencies the authority to destroy contraband drugs, a pharmacy verification and licensure system, search engine accountability, and prevention of illicit transactions.

Given the technical difficulty of reducing the risks from an enforcement point of view, the role of the consumer becomes essential. The role of the patient as an active partner in health care, and not just a passive object of diagnostic testing and medical treatment, is widely accepted. As this view is accepted, providing information to patients becomes a very crucial issue. Attempts to create some sort of labeling to distinguish trustworthy from rogue websites are valuable, but they cannot be very effective as long as people are not aware of these tools and of the risks involved in buying medication online. As described by Eysenbach [134], medicine 2.0 includes the concept of a shift from an “intermediation environment” (based on the power of the experts) to an “apomediation environment” (based on the empowerment of users). The latter is “desirable by older adolescents and adults, experienced or information literate consumers”, otherwise the risks of increased autonomy can far outweigh the benefits in a context like that of free access to drugs.

In conclusion, online pharmacies are a case where major conflicts occur between the concept of individuals being able to decide their purchases in their own interests on the one hand, and on the other the demand that the state must prevent people from harming themselves and must use public resources fairly and efficiently, as well as the value of social solidarity [23]. In order to enhance the benefits and minimize the risks of online pharmacies, a 2-level approach could be adopted. The first level should focus on policy, with laws regulating the phenomenon at an international level, filling the existing legislative vacuum, although, as stated above, this would be very difficult, costly, and only partially effective. The second level needs to focus on the individual. This approach should aim to increase health literacy, which is the foundation of critical thinking, a skill required for making appropriate health choices, recognizing risks, and making the most of the multitude of opportunities offered by the world of medicine 2.0.

**Acknowledgments**

The authors wish to thank the anonymous reviewers for their valuable comments and insightful suggestions to improve the quality of the paper.

**Authors’ Contributions**

GO participated in the conception and the design of the study, checked the collected data, analyzed and interpreted the data, and drafted the article; AM collected and assembled the data; PJS participated in the conception and the design of the study and made a critical revision of the article; UG participated in the conception and the design of the study and collection and interpretation of data, and continuously revised the article during drafting. All authors approved the final article.

## Multimedia Appendix 1

List of articles about online pharmacies without original data.

## Multimedia Appendix 2

List of articles about online pharmacies, sorted in alphabetical order according to first author; the characteristics of each study’s sample is shown in terms of year of data collection, selection method, inclusion criteria, and number of online pharmac

## Multimedia Appendix 3

Articles about case reports involving medicines bought on an online pharmacy, listed in alphabetical order according to the first author.

## Abbreviations

CASA: National Center on Addiction and Substance Abuse
FDA: Food and Drug Administration
GAO: Government Accountability Office
HINTS: Health Information National Trends Survey
HON: Health on the Net Foundation
JCAHCO: Joint Commission on Accreditation of Healthcare Organizations
MTF: Monitoring The Future
NABP: National Association of Boards of Pharmacy
NSDUG: National Survey of Drug Use and Health
PDE5Is: phosphodiesterase type 5 inhibitors
RADARS System: Researched Abuse Diversion and Addiction-Related Surveillance System
VIPPS: Verified Internet Pharmacy Practice Sites
WHO: World Health Organization
