# Supplementary material for: Quality of Online Pharmacies and Websites Selling Prescription Drugs: A Systematic Review
Source: J Med Internet Res. 2011 Sep 30;13(3):e74. doi: 10.2196/jmir.1795 (PMC3222188; doi:10.2196/jmir.1795)
Supplement: Supplementary file 1 [file jmir_v13i3e74_app1.pdf]

Appendix 1

List of articles about online pharmacies without original data

1. Atkinson H.Online Pharmacies. HealthNews 1999 Mar 25;5(4):2.

2. Baert B, De Spiegeleer B. Quality analytics of Internet pharmaceuticals. Anal Bioanal Chem. 2010 Sep;398(1):125-36.

3. Barcia SM. Internet Pharmacies: All Hype With No Help? Have Pharmacists finally given the shop away? Health Manag Technol 2000 Apr;21(4)24-25.

4. Bessel TL, Anderson JN, Silagy CA, Sansom LN, Hiller JE. Surfing, Self-medicating and safety: buying non-prescription and complementary medicines via internet. Qual Saf Health Care 2003;12:88-92.

5. Binns R, Driscoll B. The internet, pharmaceuticals and the law. Drug Discov Today 2001 May;6(9):452-453.

6. Bonakdar RA. Herbal cancer cures on the web: non compliance with the dietary supplement health and education act. Fam Med 2002 Jul-Aug;34(7):522-527.

7. Bostwick JM, Lineberry TW. Do Cheap internet drugs threaten the safety of the doctor-patient relationship? Expert Opin Drug Saf 2007;6(1):9-13.

8. Bosworth DL. Counterfeiting in global pharmaceuticals sector: its consequences and management. International Journal of Intellectual Property Management 2009;3(4):343-356.

9. Bradley C, EH Hansen, S Kooiker. European observatory on Health System and Policies Series. Regulating pharmaceuticals in Europe: striving for efficiency, equity and quality. Chap 9: Patients and their medicines. Regulating pharmaceuticals in Europe. Maidenhead, Mc Graw Hill; 2004.:158-176.

10. Bruckel K, Capozzoli EA. Internet pharmaceutical sales: attributes, concerns, and future forecast. J Hosp Mark Public Relations 2003;15(1):61-76.

11. Brushwood DB. Responsive regulation of internet pharmacy practice. Ann Health L 2001 Apr;10:75-103.

12. Cardinale V. In step with on-line pharmacies. Drug topics 1999 Aug;143(15):10.

13. Castronova JR. Operation Cyber Chase and Other Agency Efforts to Control Internet Drug Trafficking. The “Virtual” Enforcement Initiative Is Virtually Useless. J Leg Med 2006;27:207-224.

14. Chant D. Mail-Order Pharmacies. Teaching your patients to use it safely . Home healthcare Nurse 1998 July;16(7):438-442.

15. Charatan F. Kansas sues internet medicine suppliers. BMJ 1999 June;318:1720.

16. Childs M, Ellison L, Prayle D. Drugs and the Internet. New Law Journal 1998 Dec:1840-1842.

17. Clifton LBS. Internet Drugs Sales: Is It time To Welcome “Big Brother” into Your Medicine Cabinet? J Contemp Health Law Policy 2004 Spring;20(2):541-70.

18. Conlan MF. Net watchers. Drug Topics 1999;143(16):71.

19. Cook Gretchen W. “No prescription required”; online pharmacies: practical o perilous? Asthma Magazine 2003 Sept-Oct;19-22.

20. Crawford SY. Internet pharmacy: issues of access, quality , costs, and regulation. J Med Syst 2003 Feb;27(1):57-64.

21. De Clippele F. Legal aspects of on-line pharmacy. Acta chi belg 2004;104:364-370.

22. Department of Justice - Drug Enforcement Administration. Implementation of the Ryan Haight online pharmacy consumer protection act of 2008; Final Rule. Federal Register Rules and Regulation 2009 Apr;74(64):15596-15625.

23. Donaldson C. In Practice. J R Soc Promot Health 2008 Nov;128(6):284.

24. Dupuits FMHM. The Effects of the Internet on Pharmaceutical Consumers and Providers. Dis Manage Health Outcomes 2002;10(1):679-691.

25. Eysenbach G. Online prescriptions of pharmaceuticals: Where is the evidence for harm or for benefit? A call for papers and for reflection. J Med Internet Res 2001;3(1):e1.

26. Easton G. Clicking for pills. BMJ 2007 Jan;334:14-15.

27. Fogel J, Novick D. Direct-to-consumer advertisement of prescription medications over the Internet. Health Mark Q 2009;26(4):347-71.

28. Forman RF. Narcotics on the Net: The Availability of Web Sites Selling Controlled Substances. Psychiatric Service 2006 Jan;57(1):24-26.

29. Fung CH, Woo HE, Asch SM. Controversies and legal issues of prescribing and dispensing medications using the Internet. Mayo Clin Proc 2004; 79: 188–194.

30. Gallagher JC, Colaizzi JL. Issues in internet pharmacy practice. Ann Pharmacother 2000 Dec;34:1483-14.

31. Gandhi TU, Nguyen-Khoa BA. Internet pharmacies: a practical analysis of the issues. Medication and Aging 2000 Wint:75-82.

32. George C. Online healthcare: Internet pharmacies may not always be good for your health . The information society: emerging landscapes. IFIP International Conference on Landscape of ICT and Social Accountability, Turku, Finland, 2005 Jun 27-29.

33. Gersh M. Business evolution or revolution? Mail-order pharmacies in Germany. Int. J. Electronic Healthcare 2004;1(1):17-32.

34. Ghodse H. Watching internet pharmacies. Br J Psychiatry 2010 Mar;196:169-170.

35. Green K. Marketing health care products on the internet: a proposal for updated federal regulation. Am J Law Med 1998;24(2/3):365-386.

36. Greenaway NJ. Internet and European drug regulation. The Lancet 1999 Jan;353:330.

37. Grund J, Vartdal TE. Distribution of pharmaceuticals - a Norvegian logistc perspective. Pharm World Sci 2000 Jun;22(3):109-115.

38. Gupta A, Drabu S, Lather AS. Online pharmacies: Global Regulatory Perspective – September 2010. Journal of pharm pharmacol 2010;62(10):1514-1515.

39. Henney JE. Cyberpharmacies and the role of the US Food and Drug Administration. J Med Internet Res 2001;3(1):e3.

40. Henney JE. Internet Purchase of Prescription Drugs: Buyer Beware. Ann Intern Med 1999 Dec;131(11):861-862.

41. Hochberg JW. Nailing Jell-O To a Wall: Regulating Internet Pharmacies. J Health Law 2004 Summer;37(3):445-471.

42. Holmes ER, Tipton DJ, Desselle SP. The impact of the internet on community pharmacy practice: a comparison of a Delphi panel's forecast with emerging trends. Health Mark Q 2002;20(2):3-29.

43. Jackson G. Faking it: the dangers of counterfeit medicine on the internet. Int J Clin Pract 2009;63(2):181-184.

44. Jackson G, Arver S, Banks I, Stecher VJ. Counterfeit phosphodiesterase type 5 inhibitors pose significant safety risks. Int J Clin Pract. 2010 Mar;64(4):497-504.

45. Johnston JC. Life threatening intracerebral hemorrhage with isometheptene mucate, dichlorophenazine and acetaminophen combination therapy. J Forensic Leg Med 2009;16:489-491.

46. Kahan SE, Seftel AD, Resnick MI. Sidenafil and the Internet. J Urol 2000 Mar;163(3):919-923.

47. Kaye T. Mail order programs can provide savings, but HMOs need to ensure that they are actually receiving sufficient value. Mail Order Pharmacy - Savings or Added Cost? Managed Care 2003 Jun:1-5.

48. Korcok M. Pharmaceutical Industry. Internet pharmacy: the tug-of-war intensifies. Can Med Assoc J 2004 Mar;170(6):946-947.

49. Lanier WL. Near-Death Experience Delivered to Your Home by Your Friend on The Internet. Mayo Clin Proc 2004 Aug;79(8):970-982.

50. Larkin M. US online pharmacies strive for respectability. The Lancet1999 Aug; 354:782.

51. Larson BS. Medication through the internet : what clinicians and patients need to know. J of Pain & Palliat Care Pharmacother 2002;16(2); 49-57.

52. Letkiewicz S, Górski A.The potential Dual Use of Online Pharmacies. Sci Eng Ethics 2010 Mar;16(1):59-75.

53. Liang BA, Mackey T. Searching for Safety: Addressing Search Engine, Website and Provider Accountability for Illicit Online Drug Sales. Am J Law Med 2009;35(1):125-184.

54. Lorence DP. The internet and civil disobedience: examining a new form of e-health behavior. Int J Electronic Healthcare 2008;4, Nos3/4:236-243.

55. Lorman AJ. Internet pharmacies catch on with consumers, pose problems for regulators. Health Care Advisory 2000 Feb:1-4.

56. Lowes R. Are online pharmacies good for your patients-and for you? Medical Economics 2000 Jun;11:77.

57. Ma J. Lowering prescription drug prices in the United States : are reimportation and internet pharmacies the answer? Southern California Interdisciplinary Law Journal 2006;15:345-375.

58. Maddox LM. The use of pharmaceutical web sites for prescription drug information and product requests. Journal of product & Brand Management 1999;8(6):488-496.

59. Makinen MM, Rautava PT, Forsström JJ. Restrictions on import of drugs for personal use within the European single market. Eur J Pub Health 2002 Dec;12(4):244-248.

60. Martinez FJ. Acute exacerbations in idiopathic pulmonary fibrosis. Annals of Internal Medicine 2006 Feb;144(3):218-227.

61. McCarthy M. Prescription drug abuse up sharply in the USA. World Report. www.thelancet.com 2007 Vol 369.

62. Miller TE, Derse AR. Between strangers: the practice of medicine online. Health Affairs 2002 August;21(4):168-179.

63. Molzon JA.E-commerce Drug promotion and sales through the internet. 10th International Conference of Drug Regulatory Authorities (ICDRA), Hong-Kong, China, 24-27 June 2002. Proceedings:117-122.

64. Montoya ID, Jano E. The Pharmaceutical Industry. Online Pharmacies: Safety and Regulatory Considerations. International Journal of Health Service 2007;37(2):279-289.

65. Munro GS. Regulation of mail-order pharmacy. Journal Leg Med 1991;12(1):1-58.

66. Murguia E, Tackett-Gibson M, A Lessem. Real drugs in a virtual world. Drug discourse and community online. Chap 3: The new drugs internet survey: a portrait of respondents. Plymouth, Lexington Books, 2007.

67. Navarro RP. Internet pharmacies: opportunities and challenger. Pharmacy Practice. Managed Care Interface Jun 1999:64-67.

68. Nielsen S, Barratt MJ. Prescription drug misuse: Is technology friend of foe? Drug Alcohol Rew 2009 Jan;28:81-86.

69. (No Author listed). Internet open access to contraceptive methods. Contracept Technol Update 1998 Sept;19(9):120-1.

70. Oliver AJ. Internet pharmacies: regulation of a growing industry. J Law Med Ethics 2000 Spring;28(1):98-101.

71. Paulsen M. New NABP Program Combines Criteria, Inspections to Certify Online Pharmacy Quality. J Am Pharm Assoc 1999 Nov-Dec;39(6):870.

72. Pennachio DL. When patients want to buy meds online. Med Econ 2004 Sept; 3;81(17):47-9.

73. Peterson EM. Doctoring prescriptions : federal barriers to combating prescription drug fraud against on line pharmacies in Washington. Wash L Rev 2000 Oct;75:1331-1364.

74. Powell A. Benchmark Legislation: A Measured Approach in the Fight Against Counterfeit Pharmaceuticals. Hasting L J 2010;61:749.

75. Reichertz PS. Legali Issues Concerning the Promotion of Pharmaceutical Products on the Internet to Consumers. Food Drug Law J 1996;51:355-365.

76. Rogers A.EU has limited the power to curb internet drug sales. The Lancet 1997 jun;349:1859.

77. Rosenbaum DC, Shap L. Internet pharmacies. New U.S. legislation puts the issue back into the spotlight. Health Law in Canada 2006 Nov;27(2):33-36.

78. Rost KT. Policing the “wild west” world of internet pharmacies. Food Drug Law J 2000;55(4):619-639.

79. Rotheberg M, Saving from Canadian interent pharmacies are limited. Ann Intern Med 2006;144(3):224.

80. Rothstein NA. Protecting Privacy and Enabling Parmaceutical Sales on the Internet: a Comparative Analysis of the United States and Canada. Federal Communication Law Journal 2001;53(2):343-374.

81. Scaria V. Cyber-pharmacies and emerging concerns on marketing drugs online. Online J Health Allied S cs. 2003;2:1

82. Schmidt. Pills by post? German retail pharmacies and the Internet. British Food Journal 2003;105(9):618-633.

83. Scott RL. Cybermedicine and virtual pharmacies. W Va L Rev 2001 Sum;103:407-502.

84. Sellers JA. Internet pharmacies. Am J Health Syst Pharm 2000 Apr 1;57(7):643.

85. Siva Nayanah 2009. Search engines continue to advertise rogue online pharmacies. BMJ 2009;339:b3457

86. Skolnick AA. Medical News and Perspectives. Who Considers Regulating Ads, Sale of Medical Products on Internet. JAMA 1997 Dec;278(21):1723-1724.

87. Spain W. Selling drugs online: distribution-related legal regulatory issues. International Marketing Review 2001;18(4):432-449.

88. Spears C. Consumer protection: Online sale of prescription drugs to minors not unconscionable. J Law Med Ethics 2002;30(2):315-316.

89. St George B, Emmanuel JR, Middleton KL. Overseas–based online pharmacies: a source of supply for illicit drug users? MJA 2004 Feb;180:118-119.

90. Stolfi P. Caveat emptor: regulating the online medicine man in the new frontier. J Contemp Health Law Policy 2000 Winter;17(1):377-401.

91. Sweet M. Policing online pharmacies: bioterrorism meets the war on drugs. Duke L & Tech Rew 2001;0041:1-5.

92. Tarleton Landis N. Virtual pharmacies boast easy access, privacy safeguardas. Am J Health-Syst Pharm 1999 June;56:1774-1779.

93. Taylor E. Illegal human growth hormone markets. Issues in controlling online drug sales and advertising. J Leg Med 2008 Apr-Jun;29:237-253.

94. Prepared Statement of The Federal Trade Commission on "The Internet Sale of Prescription Drugs from Domestic Websites" Before the Commettee on Government Reform. United States House of Representatives. Whashinghton, D.C. 2003 March 27.

95. Thompson M. News Feature. Buying medicines in a world wide web: what is legal and what is not? Pharm J 2003 Aug 16;271(7262).

96. Trissler R. Drug and supplement sales on the Web: Novel marketing method or potential time bomb? J Am Diet Assoc 1999 Oct;99(10):1194.

97. Tuffs A. Internet sales threaten drug companies? Supremacy. BMJ 2002 Apr;324:998.

98. Ukens C. Internet pharmacies. Drug Topics 1999 May;143(10):63-67.

99. Wadman M 2010 Drug ads move online, creating a web of regulatory challenges. Nature Medicine 2010;16(1):22.

100. Ward MJ. Online pharmaceutical regulation an avenue to a safer world. J Leg Med 2003;24:77-107.

101. Weber W. German controversy over internet pharmacy. The Lancet 2000 Dec;356:1912.

102. Weiss AM. Buying prescription drugs on the Internet: Promises and Pitfalls. Cleveland Clinic Journal of Medicine 2006;73(3):282-288.

103. Weppner WG, Hollon MF, Chew LD, Larson EB. Direct-to-consumer Offers for Free and Discounted Medications on the Internet: A Content Analysis of "e-Samples". Arch Intern Med 2009;169(21):2024-2030.

104. Whitten P., Steinfield C. and Hellmich S. Ehealth: Market potential and business strategies. JCMC 6 (4) p. 1-21 July 2001

105. WHO . Information Exchange System Alert No. 122. Warning on purchase of antivirals without a prescription including via the internet. 2009 May 14.

106. WHO Pharmaceuticals and the internet Drug Regulatory Authorities' Perspective. Who 34-25 September 2001, Copenhagen, Denmark

107. Wilkinson E. Is the online drugs market putting patients at risk? NT 2006 Nov;102:46:23-24.

108. Williams BJ. On line prescriptions and drug sales: an overview of emerging issues. Hous J Health L & Pol'y 2001;1-36.

109. WJM. Online pharmacies: industry growing, but safety questions remain. WJM 2000 Dec;173:375.

110. Yoo K. Self prescribing medication: regulating prescription drug sales on the Internet. The John Marshall Journal of Computer & Information Law 2001 Fall:1-39.

111. Young D. GAO details perils of internet drug buying. Am J Health Syst Pharm 2004;61(15):1526-28.

112. Young D. VIPPS Program suspends online pharmacy. Am J Health-Syst Pharm 2001 Oct;58:1786-1788.

113. Young D. FDA clarifies importation law as Internet pharmacies proliferate. Am J Health-Syst Pharm 2003 Apr;60:729-730.

114. Zbar JD. Pharmacies surge online. Consolidation under way in turf war for repeat Net customers. Advertising Age 2000 Apr:58.

115. Zehnderl S. Swiss Community Pharmacies' on the Web and Pharmacists' Experiences with E-commerce: Longitudinal study and Internet-based questionnaire survey. J Med Internet Res 2004 Mar 3;6(1)E9.

116. Zeman SE. Regulation of online pharmacies: a case of cooperative Federalism. Speciality Digest: Health Care:9-41.

117. Zwick S. A dutch dotcom that fills prescriptions for its neighbors challenges Germany's strict regulation. Time Europe 2001;157(10).
