# Supplementary material for: Quality of Online Pharmacies and Websites Selling Prescription Drugs: A Systematic Review
Source: J Med Internet Res. 2011 Sep 30;13(3):e74. doi: 10.2196/jmir.1795 (PMC3222188; doi:10.2196/jmir.1795)
Supplement: Supplementary file 2 [file jmir_v13i3e74_app2.pdf]

Appendix 2

List of articles about online pharmacies, sorted in alphabetical order according to first author; the characteristics of each study's sample is shown in terms of year of data collection, selection method, inclusion criteria, and number of online pharmacies analysed.

First Author, Year of publication	Year(s) of data collection	Online pharmacy selection method [a]	Specific inclusion criteria for online pharmacy selection. Only websites selling:	Other specific inclusion criteria	Number of online pharmacies analysed
Arruanda, 2004 [78]	2001-2002	SE			113
Armstrong, 1999 [57]	1999	SE	sildenafil	no-prescrip-tion only	77
Bate et al., 2010 [63]	2009	VIPPS+SE	Liptor, Viagra, Celebrex, Nexium, Zoloft		55
Bessel et al., 2002 [75]	2001	SE			104
Bloom et al., 1999 [58]	1999	SE			46
Bloom et al., 2006 [89]	2003- 2004	SE		not an Internet manifesta-tion of a brick-and-mortar pharmacy	144
CASA, 2008 [79]	2008	SE	controlled substances		159
Cicero et al., 2008 [93]	2006	SE	opioid analgesics		47 / 457 [b]
European Alliance, 2008 [80]	not declared	SE			116
Eysenbach, 1999 [59]	1999	SE	Viagra		22

Forman, 2003 [69]	2003	SE	opiates	no-prescrip-tion only	53
Forman et al., 2006a [70]	2003-2004	SE	opiates	no-prescrip-tion only	25
Forman et al., 2006b [71]	not declared	SE	opiates	no-prescrip-tion only	50
Forman et al., 2006c [72]	2005	SE	opiates	no-prescrip-tion only	50
Gallagher et al., 2010 [81]	2008	SE	Viagra		44
GAO, 2000 [76]	1999	SE			190
GAO, 2004 [82]	not declared	SE			68
Gernburd et al., 2007 [61]	2006	spam			19
Gurau, 2005 [83]	not declared	SE			251
Holmes et al., 2005 [62]	2004	SE+VIPPS			64
Koong et al., 2005 [14]	not declared	SE+ephar-macy-finder			10
Kunz et al. 2010 [65]	not declared	SE+Top 100 Retailers			16
Kuzma, 2011 [60]	not declared	SE			60
Levaggi et al., 2009 [95]	2007	SE			100 [c]
Littlejohn et al., 2005 [84]	not declared	SE			35
Mahé et al., 2009 [94]	2008	SE	psoriasis medicines		21
Mainous et al., 2009 [73]	2008	SE	antibiotics	no-prescrip-tion only	138

Makinen et al., 2005 [27]	2000-2001	SE		US- and Europe-based only	17
Memmel et al., 2005 [68]	2004	SE	Contra-ceptives		4
NABP, 2010 [10]	2010	not declared			5859
Orizio et al. 2009a [85]	2007	SE			118
Orizio et al., 2009b [90]	2008	SE		only online pharmacies using online question-naires	57 [d]
Orizio et al., 2010 [86]	2008	SE			175
Peterson, 2001 [77]	2000	SE		US-based only	33
Peterson et al., 2003 [92]	2001	SE		US- based only	29 [e]
Quon et al., 2005 [64]	2004	pharmacy checker		US- and Canadian-based only [f]	12
Raine et al., 2009 [87]	not declared	SE	analgesics	delivery to UK only	46
Schifano et al., 2006 [88]	2003-2005	SE	Dextropro-poxyphene		20
Soares Gondim et al., 2007 [91]	2005	not declared		only Brazil-based	18
Tsai et al., 2002 [74]	2001-2002	SE	Cyproflox-acine	no-prescrip-tion only	59
Veronin et al., 2007 [66]	2006	SE		Canadian-based only	4
Wagner et al., 2001 [67]	1999-2000	specifc method[g]	Parkinson's medicines	US- based only	4

(a). The method of online pharmacy selection was: SE, search engine; VIPPS, pharmacychecker and epharmacyfinder site: online pharmacies listed in the VIPPS Verified Internet Pharmacy Practice

Site, at Pharmacychecker.com, in the Best Online Pharmacy Guide at epharmacyfinder.com ; and/or in the Stores Top 100 Retailers (Schulz DP. Top 100 retailers: the nations's retail power players. Stores 2008;90:19).

(b) They randomly chose about 1/10 circa of the 457 websites found for a further analysis of the characteristics.

(c) A sub-analysis by Orizio et al. 2009a [85], based on the evaluation of pricing and marketing aspects.

(d) A sub-analysis by Orizio et al. 2009a [85], based to the evaluation of online questionnaires.

(e) A follow-up to Peterson's 2001 [77] study: 29 of the 33 online pharmacies were still working.

(f) Method: 12 Canadian-based Internet pharmacies (identified from the Pharmacychecker.com list) and 3 US-based drug chain pharmacies.

(g) Method: Comparison of three Internet pharmacies websites associated with US retail chain pharmacies and one independent Internet pharmacy website associated with a randomly chosen independent retail pharmacy in New Jersey.
