# Supplementary material for: Quality of Online Pharmacies and Websites Selling Prescription Drugs: A Systematic Review
Source: J Med Internet Res. 2011 Sep 30;13(3):e74. doi: 10.2196/jmir.1795 (PMC3222188; doi:10.2196/jmir.1795)
Supplement: Supplementary file 3 [file jmir_v13i3e74_app3.pdf]

Appendix 3

Articles about case reports involving medicines bought on a online pharmacy, listed in alphabetical order according to the first author.

First Author, Year of publication	Year of case	Country of occurrence	Age of case	Gender of case	Medicine involved
Barnes et al., 2003 [119]	2003	US	40	female	haloperidol, bentazepam
Dalal et al., 2004 [122]	2004	US	43	female	caesium chloride
Eleid et al., 2010 [121]	2010	US	51	male	carisoprodol
Hainer et al., 2000 [123]	2000	US	55	male	hydrazine sulfate
Levesque, 2004 [125]	2004	US	67	male	diphenhydramine hydrochloride, ephedrine and various kinds of “tranquillizers”
Lineberry et al., 2004 [124]	not declared	US	35<br>37<br>42<br>29	male<br>male<br>male<br>female	amitriptyline<br>opiates<br>tramadol<br>hydrocodone
Neuberg et al., 2009 [120]	2009	US	56	female	Armour thyroid (dessicated porcine thyroxine [T4] and triiodothyronine [T3])
Pirola et al., 2010 [126]	2010	Italy	34	male	Anabolic steroids
Romero et al., 2004 [118]	2004	US	37	female	Butalbital (Fioricet)
Solomon et al., 2002 [128]	2002	UK	41	male	sildenafil citrate

Thurairajah et al., 2005 [127]	2005	UK	57	female	Orlistat (Xenical)
Winickoff et al., 2010 [117]	2010	US	18	female	γ-butyrolactone
